# Individual Dietary Consultation Utilization and Patient-Reported Experiences Among People with Type 2 Diabetes in Israel: A Cross-Sectional Study

**DOI:** 10.3390/nu18060990

**Published:** 2026-03-20

**Authors:** Michal Kasher Meron, Adi Givati, Mahmoud Jomah, Idit Dotan, Talia Diker Cohen, Liat Barzilay-Yoseph, Sofia Shapira, Nuha Younis Zeidan, Vered Kaufman-Shriqui, Ofra Kalter-Leibovici, Pnina Rotman-Pikielny

**Affiliations:** 1 Institute of Endocrinology, Meir Medical Center, Kfar Saba 4428164, Israel; adigi1@clalit.org.il (A.G.); liat.barzilay-yoseph@clalit.org.il (L.B.-Y.); sofiabr1@clalit.org.il (S.S.); pnina.rotman@clalit.org.il (P.R.-P.); 2 School of Medicine, Gray Faculty of Medical and Health Sciences, Tel-Aviv University, Tel-Aviv 6997801, Israel; 3 Gertner Institute of Epidemiology and Health Policy Research, Sheba Medical Center, Ramat Gan 5262100, Israel; ofral@gertner.health.gov.il; 4 Internal Medicine Department, Meir Medical Center, Kfar Saba 4428164, Israel; mahmudjum@clalit.org.il; 5 Institute of Endocrinology, Diabetes and Metabolism, Beilinson Hospital, Rabin Medical Center, Petah Tikva 4941492, Israel; iditdo@clalit.org.il (I.D.); taliadc@clalit.org.il (T.D.C.); 6 Clalit Health Services, Tel Aviv 6927022, Israel; nuhaze1@clalit.org.il; 7 Department of Nutrition Sciences, Ariel University, Ariel 4070000, Israel; veredks@ariel.ac.il; 8 Dina Recanati School of Medicine, Reichman University, Herzliya 4610101, Israel

**Keywords:** diabetes mellitus, type 2, medical nutrition therapy, diet, Mediterranean, patient acceptance of health care, health services utilization, dietitians, health knowledge, attitudes, practice, Israel

## Abstract

**Objectives**: To describe the utilization patterns and patient perceptions of individual dietary consultations among people with type 2 diabetes in Israel, and to examine the association between dietary consultation attendance and adherence to the Mediterranean diet. **Methods**: This cross-sectional study enrolled adults with type 2 diabetes from a specialty diabetes clinic in Israel between July 2022 and May 2023. Participants completed structured interviews in which they were asked to report their perceptions of various diabetes management components, their sources of dietary information, and—among those who had previously attended dietary consultations—their satisfaction with specific aspects of the consultation experience. Medical records were reviewed to determine attendance at dietary consultations. Adherence to the Mediterranean diet was measured using the validated I-MEDAS 17-item questionnaire. Multivariable logistic regression was used to examine the association between attendance at dietary consultation within the past 12 months and adherence to the Mediterranean diet, adjusting for age, sex, socioeconomic status, and obesity (BMI ≥ 30 kg/m^2^). **Results**: Overall, 134 patients were interviewed. Their mean age was 69.8 ± 10.7 years, mean diabetes duration was 19.1 ± 9.7 years, and 96.3% were Jewish. Only 29.1% attended a dietary consultation within the past 12 months, and 52.2% had at least one consultation over the preceding 5 years. While 79.9% of participants rated maintaining normal weight and 78.4% rated taking medications as “very helpful” for diabetes control, only 29.9% reported that regular dietitian visits would be “very helpful.” Most participants (74.6%) were unable to name a specific dietary pattern they were following. Among those who recalled ever attending dietary consultations, most reported in interviews that recommendations were culturally aligned with their preferences. No association was found between recent attendance at dietary consultations and adherence to the Mediterranean diet (adjusted OR 1.03, 95% CI 0.39–2.74). **Conclusions**: Despite having accessible and affordable individual dietary consultations, the utilization of this service remains low, and patient-reported benefit limited. These exploratory findings point to perception-based barriers to engagement that warrant further investigation.

## 1. Introduction

Nutrition therapy is recognized by diabetes professional guidelines as an essential component of diabetes self-management education and support (DSMES) [1,2]. Registered dietitians delivering diabetes-specific medical nutrition therapy should regularly assess individual nutritional needs, provide evidence-based nutrition education and counseling, and collaboratively establish patient-specific goals [1,3]. Attendance at dietary consultations is associated with lower HbA1c and reduced weight [4,5]. Despite these demonstrated benefits, the real-world utilization of dietary consultation services remains suboptimal globally. Studies from multiple countries report that fewer than half of individuals with type 2 diabetes attend structured dietary education programs or individual consultations [6,7,8,9].

In Israel, individual dietary consultations are delivered nationwide by registered clinical dietitians and reimbursed by national health insurance for up to 14 visits annually with minimal copayment [10]. Data on utilization patterns and patient experiences with dietary consultations among people with type 2 diabetes is limited.

While determinants of attendance at diabetes self-management education programs have been examined in several countries [6,7,9,11], data on the utilization of individual dietary consultation and how patients perceive these services remain scarce, particularly in the context of a universal healthcare system where structural and financial access to these services is not a limiting factor. The aim of this cross-sectional study was to describe the utilization and perceptions of dietary consultations among individuals with type 2 diabetes attending a specialty diabetes clinic in Israel. A secondary objective was to examine the association between dietary consultation attendance and adherence to the Mediterranean diet. This dietary pattern is recommended for diabetes management [1,2] and has demonstrated benefits for cardiovascular disease prevention [12,13] and glycemic control [14]. The Mediterranean diet is culturally accepted in Israel and endorsed by the Israeli Ministry of Health [15].

## 2. Subjects and Methods

### 2.1. Study Design and Population

We conducted a cross-sectional study at a single diabetes clinic at Meir Medical Center, which is part of Clalit Health Services. It is a tertiary medical center serving approximately 1 million residents of a mainly urban area.

The inclusion criteria were age > 18 years and a diagnosis of type 2 diabetes. People who were diagnosed less than six months prior to the diabetes clinic visit, pregnant women, and people who were unable to converse in Hebrew or provide informed consent were excluded. Participants were recruited from those attending the diabetes clinic on randomly selected days between July 2022 and May 2023. For the second aim, based on a sample size calculation to detect a correlation coefficient of 0.25 with a type-I error of 5% and a power of 80%, we determined that 123 participants were required. To account for potential data incompleteness, we aimed to recruit 134 participants (approximately 9% above target). Of the 167 people we approached, 134 agreed to participate and completed a 30 min interview with a trained interviewer. Interviewers underwent a one-hour standardized training session prior to data collection, during which they were instructed to adhere to the exact wording of the survey instrument, and were blinded to participants’ medical records throughout the interview process. The remaining individuals either declined participation or could not be interviewed due to clinic scheduling constraints and interviewer availability. We did not collect data on non-participants; therefore, selection bias cannot be ruled out. However, the high participation rate (80%) suggests reasonable representativeness of clinic attendees. The study protocol, including verbal informed consent, was approved by the Meir Medical Center Ethics Committee (MMC-0076-22).

### 2.2. Study Variables

#### 2.2.1. Questionnaire Development and Content

While several validated instruments exist to assess diabetes self-care activities and adherence (such as the Diabetes Self-Management Questionnaire and the Summary of Diabetes Self-Care Activities), these tools primarily measure the frequency of self-care behaviors rather than patients’ subjective perceptions of the relative impact or helpfulness of specific diabetes management components. For the purposes of this study, we sought to specifically capture patients’ perceptions regarding which aspects of diabetes care they perceived as most beneficial to their overall diabetes management, with particular interest in understanding how dietary consultation was valued relative to other components of care (e.g., medications, glucose monitoring, physical activity counseling, physician visits).

A structured questionnaire was therefore developed de novo for this study and administered by interviewers using a standardized protocol. Participants rated how helpful each of the following components would be in achieving better diabetes control (defined as better glucose levels on blood tests): (1) physical activity (walking, gym), (2) regular visits with a nutritionist, (3) stress management (not getting upset), (4) maintaining normal body weight, (5) following a diabetes-specific diet, and (6) taking prescribed medications (oral or injectable). Response options were presented on a 4-point scale: “would be very helpful”, “somewhat helpful”, “not so helpful”, and “not helpful at all”. The complete questionnaire (translated from Hebrew) is provided as Supplementary Materials.

Additionally, participants were asked whether they adhered to a specific dietary pattern, with options to select from multiple predefined patterns or specify their own under “other”. We assessed the participants’ sources of diabetes dietary education or guidance using an open-ended approach, wherein interviewers asked participants to spontaneously name their sources of dietary information and documented the responses without prompting with a predetermined list. Responses were subsequently categorized by the interviewer into predefined groups (e.g., registered dietitians, physicians, family/friends and internet/social media).

#### 2.2.2. Assessment of Dietary Consultation Attendance

Dietary consultation attendance was assessed through multiple complementary methods. Dietary consultation attendance included individual in-person or telehealth sessions with registered dietitians documented in the health maintenance organization (HMO) electronic health record system in the 12 months preceding the study. Group education sessions address multiple aspects of diabetes self-care beyond dietary guidance and are not documented in the HMO database; they were therefore not included in the attendance measure. Since the observed attendance was highly skewed, with the majority of participants (70.9%) having no recorded consultations and very few having attended more than once, attendance was analyzed as a binary variable (any consultation vs. none) in the primary analysis. To provide a broader context of long-term utilization patterns, data were also extracted from the HMO data center to quantify the total number of dietary consultations attended by each participant over the 5-year period, 2019 to 2023.

#### 2.2.3. Patient-Reported Outcome Measures

In addition to an objective chart review, participants were asked whether they had ever attended a dietary consultation (yes/no). For those who attended, we developed a structured questionnaire to assess patient perceptions and satisfaction regarding specific aspects of dietary consultations, including cultural appropriateness, personalization to individual preferences, and the palatability of the recommendations. Although validated tools for assessing patient satisfaction exist, such as the Client Satisfaction Questionnaire-8 (CSQ-8) and the Diabetes Treatment Satisfaction Questionnaire (DTSQ), these instruments assess general treatment satisfaction rather than the specific dimensions of dietary consultation quality we sought to evaluate.

The questionnaire assessed: (1) Whether the dietitian spoke their language, (2) whether dietary recommendations were affordable, (3) whether dietary recommendations were adjusted to their culture and family context, (4) whether the recommended foods were tasty, (5) whether the dietary plan was satiating, and (6) whether the recommendations took into account their personal food preferences. Each item was presented as a yes/no question assessing satisfaction with a specific aspect of the consultation experience (Supplementary Materials). Participants who responded negatively were then asked to rate the degree to which that aspect interfered with their consultation experience on a Likert-type scale. As virtually all participants reported satisfaction across all dimensions, responses were analyzed and reported as binary outcomes.

Face-validity of the questionnaire was established through expert review by a multidisciplinary panel consisting of endocrinologists, epidemiologists, and registered dietitians, who assessed the questionnaire for clarity, relevance, and appropriateness of content in the Israeli healthcare context. Individual questionnaire items were analyzed descriptively rather than combined into composite scores, allowing for the detailed characterization of patient perceptions across different aspects of dietary consultation experiences. Given that the instrument was not formally validated or pilot-tested in the target patient population, findings derived from this questionnaire should be interpreted as exploratory and descriptive.

#### 2.2.4. Adherence to the Mediterranean Diet

The main outcome variable was Mediterranean diet adherence, measured by the 17-item I-MEDAS questionnaire [13], an adaptation of the 14-item MEDAS questionnaire from the Spanish PREDIMED study [16,17]. In the original MEDAS questionnaire, participants were asked to estimate their daily or weekly intake of various foods, earning one point per item if predefined criteria were met. For instance, two servings or more of non-starchy vegetables per day, with one serving defined as 200 g, earned one point. The I-MEDAS includes food items which are in accordance with the Mediterranean diet principles and are widely consumed in Israel (e.g., tahini, hummus and low-fat dairy) instead of food items that appeared in the original MEDAS and are rarely consumed locally (e.g., shellfish and savory tomato sauce). The I-MEDAS has been previously validated and shown to correlate with all-cause mortality in a large Israeli cohort study [13].

The participants’ electronic medical records were thoroughly reviewed to collect socio-demographic, clinical, biochemical, and administrative data. Routine laboratory tests taken up to 6 months prior to the interview visit were recorded. Socioeconomic status was determined using the Israeli Socioeconomic Score (1–10), periodically published by the Central Bureau of Statistics [18], where higher scores indicate greater affluence. This index incorporates various indicators such as average income, education level, employment rate, standard of living, and demographic characteristics.

### 2.3. Statistical Analysis

In this cohort, Mediterranean diet adherence scores were relatively high and displayed a narrow normal distribution. Therefore, the outcome continuous score was dichotomized into the highest tertile versus the lower two tertiles, to better capture potential differences in the “high score” subgroup while still retaining a sufficiently large comparison group. While dichotomization reduces statistical power compared to continuous analysis, it was the most clinically meaningful approach given the narrow distribution. Missing data were minimal (<1%): One participant had incomplete I-MEDAS data and was excluded from the dietary adherence analysis (listwise deletion).

Baseline characteristics are presented as means and standard deviations for continuous variables, and as frequencies and percentages for categorical variables. For categorical variables, Chi-square tests were used. For continuous variables, normality was assessed; normally distributed variables were compared using independent samples *t*-test, while non-normally distributed variables were analyzed using the Kruskal–Wallis test.

The primary analysis used multivariable logistic regression to assess the association between dietary consultation attendance and high adherence to the Mediterranean diet (upper tertile compared to the lower two tertiles), with adjustments for age, sex, and socio-economic status, and obesity (BMI ≥ 30 kg/m^2^). Additional clinical variables including HbA1c, diabetes duration, and insulin use were not included as covariates, as they did not differ significantly between attenders and non-attenders (Table 1), making them unlikely confounders. Odds ratios (OR) were calculated with 95% confidence intervals (CIs). Logistic regression model assumptions were verified, including absence of multicollinearity among predictors (variance inflation factor < 2.0 for all variables). The goodness of fit of the model was evaluated with the Hosmer–Lemeshow statistic. Data were analyzed using IBM SPSS statistics software version 29.0 (SPSS Inc., Chicago, IL, USA). A two-sided *p*-value < 0.05 was considered statistically significant.

## 3. Results

The study cohort included 134 patients, of whom 61 (45.5%) were male and 129 (96.3%) were Jewish. Mean age was 69.8 ± 10.7 years, mean BMI was 29.3 ± 4.8 kg/m^2^, and mean diabetes duration was 19.1 ± 9.7 years. Mean hemoglobin A1c was 7.1 ± 1.1%, and mean LDL cholesterol was 68.7 ± 29.0 mg/dL (Table 1).

**Figure 1:**
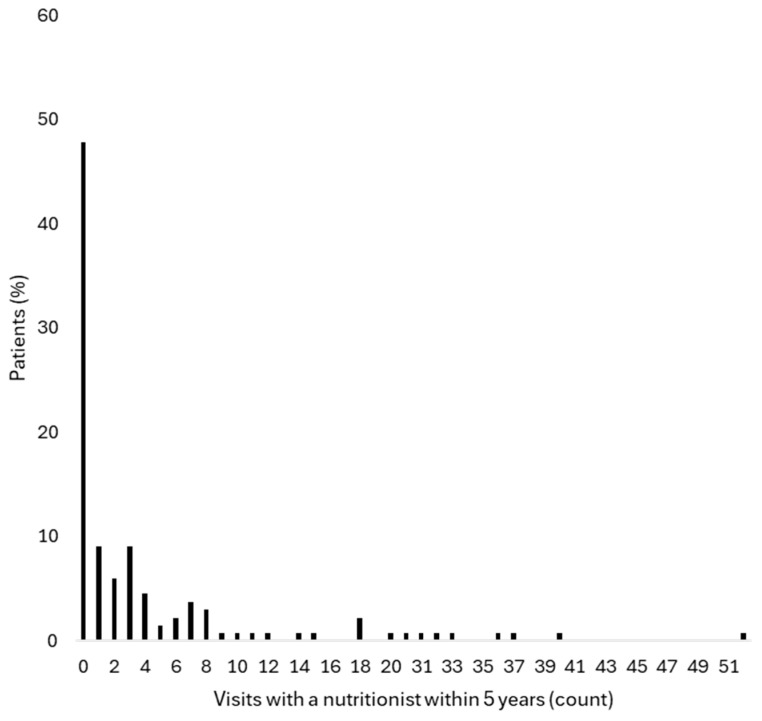
The distribution of dietary consultation attendance over a 5-year period (2019–2023). The percentage of participants by number of dietary consultation visits recorded in the electronic health record during the 5-year period preceding the study (2019–2023).

Thirty-nine participants (29.1%) had a recorded dietary consultation within the 12 months preceding the interview. Patient characteristics did not differ significantly between those who had and had not attended dietary consultations within this timeframe (Table 1). Over the 5-year period prior to the study, 70 participants (52.2%) had at least one recorded dietary consultation (Figure 1).

When asked about dietary pattern adherence, 100 participants (74.6%) reported not following any specific diet, 10 (7.5%) reported following a named dietary pattern (vegetarian, vegan, or ketogenic), and 24 (17.9%) reported adherence to an unspecified dietary pattern (Table 2). When asked to describe this pattern, most indicated they were following recommendations from a physician or dietitian but could not name a specific diet type.

When asked to rate the helpfulness of various interventions for glucose control, 107 participants (79.9%) stated that maintaining normal body weight and 105 (78.4%) stated that taking anti-diabetic medications would be “very helpful.” Eighty participants (59.7%) rated eating a diabetes-appropriate diet as “very helpful,” yet only 40 (29.9%) reported that regular visits with a dietitian would be “very helpful” in achieving glucose control (Table 3).

**Figure 2:**
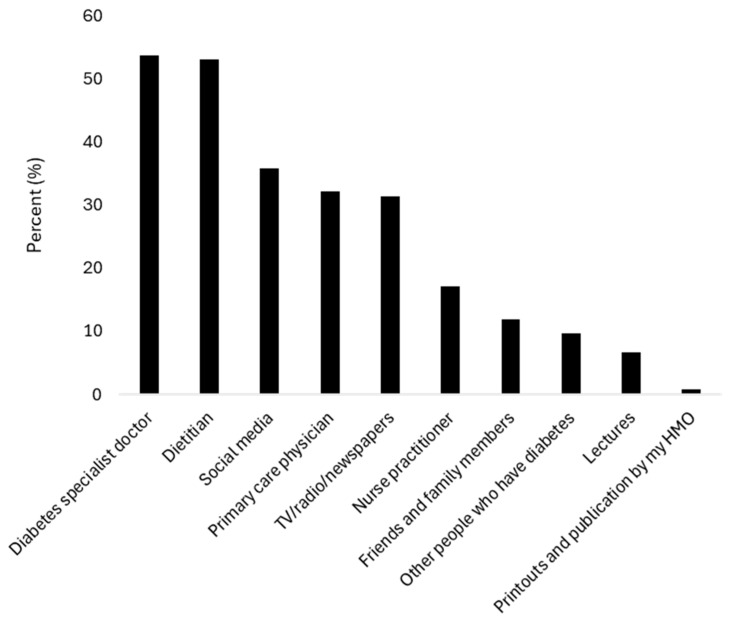
Patient-reported sources of nutritional guidance. Participants could name multiple sources. Categories include healthcare professionals (diabetes specialists, dietitians, primary care physicians) and non-professional sources (social media, friends/family). HMO, health maintenance organization. *n* = 134.

When participants were asked about their sources of dietary education or guidance, 72 (53.7%) named their diabetes specialist, 71 (53.0%) named their dietitian, and 43 (32.1%) named their primary care physician. Non-professional sources were also commonly cited, with 48 (35.8%) reporting social media and 16 (11.9%) reporting friends (Figure 2).

**Figure 3:**
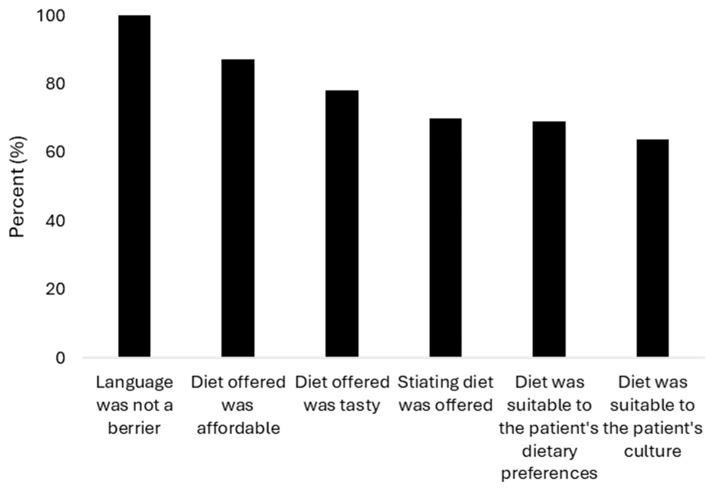
Patient-reported appropriateness of previous dietary consultations. Percentage of patients responding ‘yes’ to each patient-reported outcome question among the 110 participants who reported ever having attended a dietary consultation. All participants indicated that language was not a barrier to communication.

In the perception questionnaire, 110 participants recalled ever having attended a dietary consultation. All indicated that language was not a barrier, with most reporting that the dietary recommendations were culturally appropriate, affordable, aligned with their personal preferences, satisfying in terms of satiation, and acceptable in taste (Figure 3).

Among the 133 participants who completed the I-MEDAS questionnaire (one participant had incomplete data), scores had a mean of 11.0 ± 2.1 out of 17 possible points. The distribution was normal with limited variance, with the highest tertile comprising scores of 13 or above. In multivariable logistic regression adjusted for sex, age, socioeconomic status, and BMI, there was no significant association between documented attendance at dietary consultation within the past 12 months and achieving an I-MEDAS score in the highest tertile (adjusted odds ratio 1.03, 95% CI 0.39–2.74, Table 4).

## 4. Discussion

In this single-center survey, documented attendance records showed a relatively low utilization of individual dietary consultations among patients with type 2 diabetes. While some patients identified dietitians as a primary source of dietary information, they also reported relying on a diverse range of professional and non-professional sources for nutritional guidance. Most participants recalled having prior experience with dietitian consultations and self-reported on the perception questionnaire that recommendations were culturally appropriate and aligned with their personal preferences. Although the majority recognized the importance of dietary adherence in diabetes management, most reported on the perception questionnaire that consulting with a dietitian would not be highly helpful for their diabetes control. Finally, there was no association between attendance at dietary consultations within the past 12 months and adherence to the Mediterranean diet.

Our findings add to the limited evidence on dietary consultation utilization in Israel, indicating that sporadic or one-time attendance is the dominant pattern. Given that our cohort comprised specialty clinic patients with complex diseases and high healthcare engagement, one might expect utilization rates to exceed those of the general diabetes population. Yet rates were remarkably close to those reported among primary care patients with type 2 diabetes in Israel (25.8% over two years) [10], suggesting that low utilization is a consistent pattern across care settings.

International data similarly demonstrate low engagement with dietary education and diabetes self-management education and support (DSMES) programs, though reported rates vary across healthcare settings. Attendance rates range from less than 10% in the UK [19] to between 6.5% and 50% across different US healthcare settings [6,9], with more than two-thirds of patients in Germany reporting never attending a structured program [7]. This variability likely reflects differences in healthcare settings, insurance coverage, program availability, and patients’ health beliefs.

Notably, patient characteristics including metabolic parameters and BMI did not differ significantly between those who attended dietary consultations and those who did not. This finding is not unexpected given the sporadic and infrequent nature of attendance documented in our cohort; a single or occasional consultation is unlikely to produce sustained metabolic change [20,21].

In Israel, medical nutrition therapy is part of the basic basket of health services, available for a modest quarterly copayment, with patients entitled to up to 14 registered dietitian visits per year [10]. The Israeli national diabetes program, launched in 2019, has further promoted diabetes education and encouraged the inclusion of diabetes educators, though most patient education continues to be delivered by registered dietitians or nurses [22]. Despite this accessibility and recent efforts to expand services, utilization remains low, suggesting that barriers to engagement extend beyond financial and structural access.

Notably, in the exploratory perception data, a disconnect was observed between patients’ recognition of diet’s importance and their limited perceived value of dietitian consultations. This disconnect was unexpected given that most participants recalled these consultations as culturally appropriate and well-aligned with their personal preferences. This pattern has been documented in other settings. In Germany, the main reasons for non-participation were patients’ personal perception that DSME was not necessary [7]. In a systematic review by Horigan et al., patients perceived no benefit from attending such programs or felt they had sufficient knowledge already [11]. Small qualitative studies in the UK [23] and Canada [24] identified a gap between patients’ expectations and available nutritional and self-care education services. Patients desired more personalized instruction, multidisciplinary follow-up, and progress monitoring. People with long-standing diabetes, stable glucose control, and relatively high dietary adherence as observed in our cohort, may additionally perceive less value in attending dietary consultations, though this hypothesis requires further investigation.

Our findings suggest that the perceived appropriateness and cultural sensitivity of individual consultations do not necessarily translate into sustained engagement or belief in ongoing value. Notably, while most participants reported not following a specific dietary pattern, 17.9% indicated they were following recommendations from healthcare providers without being able to name the pattern. This suggests potential challenges in translating dietary education into concrete, memorable dietary frameworks that patients can articulate and sustain. The multiplicity of information sources may present challenges in integrating consistent dietary guidance, though whether this contributes to reduced engagement with formal consultations remains to be investigated.

We found no association between recent attendance at dietary consultation and adherence to the Mediterranean diet. The PREDIMED-Plus trial demonstrated that intensive, ongoing structured dietary consultations significantly improved adherence to the Mediterranean diet [25]. However, this carefully controlled research intervention in Spain involved frequent contact with trained dietitians, systematic behavioral support, and the provision of key Mediterranean diet foods, a level of intensity and structure rarely replicated in routine clinical practice. Real-world evidence showed mixed results: Attending the DSMES program was associated with some self-care behaviors but not with certain dietary habits in Germany [26,27], and meta-analyses indicate that educational interventions without the direct provision of food yield limited dietary behavioral change [28], with benefits waning over time [20,21]. Our findings align with this pattern, as dietary consultations were infrequent and sporadic, and recent attendance was not associated with dietary adherence.

These findings have important implications for diabetes nutritional care in Israel and beyond. While low attendance at DSMES programs has been documented across multiple healthcare systems, individual dietary consultation—a distinct and increasingly emphasized component of personalized diabetes care [1]—has received little specific attention in the literature. The exploratory findings suggest that even when individual dietary consultations are accessible, affordable, and recalled as culturally appropriate, utilization remains low and patient-reported benefit limited, pointing to the need to better understand and address perception-based barriers to engagement. Future research should investigate patients’ expectations from nutritional consultations and identify intervention characteristics—including content, structure, and delivery approaches—that promote sustained engagement. As diabetes is a chronic progressive disease, patient expectations and nutritional needs may evolve over time, making longitudinal assessment essential. Such investigations could inform the development of patient-relevant, more effective and adaptable strategies for diabetes nutritional care.

Several limitations should be acknowledged. First, this was a single-center study conducted in a diabetes specialty clinic with limited representation of minority populations, younger ages, and individuals from lower socioeconomic backgrounds. Recruitment from a specialty clinic may have introduced selection bias in either direction, toward higher utilization given greater disease complexity and healthcare engagement, or toward lower perceived benefit given long-standing, well-controlled diabetes. Second, the tool used to assess patient perceptions and satisfaction with dietary consultations was not formally validated or pilot-tested with patients, and responses may have been subject to recall bias and social desirability bias. Third, the cross-sectional design precludes causal inference between attendance and dietary adherence. Fourth, the timing of dietary consultations within the 12-month exposure window was not captured, precluding conclusions about the temporal relationship between consultation attendance and dietary behavior. Fifth, the overall high dietary adherence scores with limited variability may have reduced the ability to detect differences between groups. Furthermore, the study may have been underpowered to detect a clinically meaningful association, as reflected by the wide confidence interval around the adjusted odds ratio (0.39–2.74). Lastly, we assessed dietary adherence using the I-MEDAS screener, which measures adherence to the Mediterranean diet but does not capture other important aspects of dietary self-management, such as meal planning, portion control, and hypoglycemia management that may be influenced by dietary consultations.

## 5. Conclusions

This study from an Israeli diabetes specialty clinic observed a modest utilization of individual dietary consultation services despite accessibility and affordability. Fewer than half of participants reported dietitian consultations as highly beneficial for their diabetes management. While caution is warranted given the single-center design and the exploratory nature of the patient perception questionnaire, these findings underscore the importance of understanding patients’ perspectives and expectations to enhance the engagement and clinical impact of diabetes nutritional care in Israel.

## Figures and Tables

**Table 1:** Baseline characteristics of study participants by attendance at dietary consultation.

Characteristic	Total *n* = 134 (100%)	No Dietary Consultation Attendance *n* = 95 (70.9%)	Dietary Consultation Attendance *n* = 39 (29.1%)	*p*-Value
Age, years, *n* (%)	69.8 ± 10.7	70.1 ± 10.1	69.2 ± 12.3	0.65
Male sex, *n* (%)	61 (45.5%)	47(49.5%)	14 (35.9%)	0.15
Ethnicity, Jewish, *n* (%)	129 (96.3%)	92 (96.8%)	37 (94.9%)	0.59
Socioeconomic status *	7.8 ± 1.1	7.8 ± 1.1	7.9 ± 1.2	0.84
Diabetes duration (years)	19.1 ± 9.7	19.4 ± 7.9	18.3 ± 13.2	0.62
Anti-diabetic drugs, *n*	2.8 ± 1.1	2.9 ± 1.0	2.6 ± 1.4	0.27
Insulin use, *n* (%)	56 (41.8%)	41 (43.2%)	15 (38.5%)	0.62
Smoking, yes (%)	11 (8.2%)	9 (9.5%)	2 (5.1%)	0.41
Employed, yes (%)	46 (34.3%)	32 (33.7%)	14 (35.9%)	0.81
Lives alone, yes (%)	25 (18.7%)	18 (18.9%)	7 (17.9%)	0.89
Body mass index (kg/m^2^)	29.3 ± 4.8	29.7 ± 4.8	28.2 ± 4.5	0.12
Systolic blood pressure (mmHg)	137.4 ± 18.2	139.5 ± 19.5	132.2 ± 13.6	0.17
Diastolic blood pressure (mmHg)	71.9 ± 12.2	72.5 ± 12.6	70.4 ± 11.2	0.55
Glucose (mg/dL)	132.7 ± 41.6	132.2 ± 43.4	134.1 ± 37.3	0.82
HbA1c (%)	7.1 ± 1.1	7.2 ± 1.1	7.0 ± 1.1	0.49
Creatinine (mg/dL)	0.97 ± 0.66	0.99 ± 0.60	0.93 ± 0.41	0.58
Total cholesterol (mg/dL)	143.4 ± 35.7	142.2 ± 34.7	146.4 ± 38.2	0.54
Triglycerides (mg/dL)	159.7 ± 125.3	153.2 ± 89.2	175.6 ± 186.3	0.35
HDL (mg/dL)	46.1 ± 13.6	46.8 ± 14.5	44.6 ± 11.1	0.40
LDL (mg/dL)	68.7 ± 29.0	67.5 ± 26.9	71.6 ± 33.9	0.48
Urine albumin/creatinine (U)	16.3 (38.4)	14.5 (37.6)	22.0 (33.2)	0.27
I-MEDAS score **	11.0 ± 2.1	10.8 ± 2.1	11.3 ± 2.1	0.23

Data are presented as mean ± standard deviation, median (interquartile range) or *n* (%). Dietary consultation attendance refers to documented visits within 12 months prior to study enrollment. Categorical variables compared using Chi-square test; continuous normally distributed variables compared using independent samples *t*-test; non-normally distributed continuous variables compared using Kruskal–Wallis test. * Socioeconomic status ranges between 1 and 10. ** Dietary adherence score range is 0 to 17 points.

**Table 2:** Patient self-reported adherence to structured dietary patterns.

Dietary Pattern	*n* (%)
Vegetarian	5 (3.7)
Keto	4 (3.0)
Vegan	1 (0.7)
Paleo	0 (0)
Intermittent fasting	0 (0)
Point counting	0 (0)
Calorie counting	0 (0)
Other	24 (17.9)
None	100 (74.6)

**Table 3:** Diabetes management strategies rated very helpful by patients.

What Would Help You Control Your Glucose Levels?	Count (%)
To maintain normal body weight	107 (79.9)
To take the pills or injections the physician has prescribed	105 (78.4)
To exercise (going to the gym, walking, etc.)	81 (60.4)
Not to get upset (less stress)	81 (60.4)
To eat a diet appropriate for patients with diabetes	80 (59.7)
To have regular visits with a dietitian	40 (29.9)

Participants rated each component on a 4-point scale: ‘Would be very helpful’, ‘somewhat helpful’, ‘not so helpful’, and ‘not helpful at all’. The data shown represents those who selected ‘very helpful’.

**Table 4:** Multivariable analysis of the association between attendance at dietary consultation and adherence to the Mediterranean diet.

	OR	95% CI	*p*-Value
Age, y	0.96	0.93–1.00	0.07
Sex, male	1.35	0.56–3.25	0.51
Socioeconomic status, point	1.07	0.74–1.56	0.72
obesity (BMI ≥ 30 kg/m^2^)	0.94	0.38–2.29	0.89
Dietary consultation within 12 M	1.03	0.39–2.74	0.95

High adherence to the Mediterranean diet was defined as an I-MEDAS score in the highest tertile (≥13 points). Model adjusted for age, sex, socioeconomic status, and obesity (BMI ≥ 30 kg/m^2^). M, months; OR = odds ratio; CI = confidence interval.

## Data Availability

The datasets generated and analyzed during this study are not publicly available due to informed consent restrictions. However, anonymized data may be available from the corresponding author upon reasonable request and subject to approval by the local ethics committee.

## Supplementary Materials

The following supporting information can be downloaded at: https://www.mdpi.com/article/10.3390/nu18060990/s1, Questionnaire: Dietary consultation—patient perspective and reported experience.
