# Supplementary material for: Individual Dietary Consultation Utilization and Patient-Reported Experiences Among People with Type 2 Diabetes in Israel: A Cross-Sectional Study
Source: Nutrients. 2026 Mar 20;18(6):990. doi: 10.3390/nu18060990 (PMC13029634; doi:10.3390/nu18060990)
Supplement: Supplementary file 1 [file nutrients-18-00990-s001.zip › nutrients-4171441-supplementary.pdf]

Supplementary Materials

Dietary consultation – patient perspective and reported experience

(Translated from Hebrew)

Section 1: Perceptions of diabetes management components

Please rate for each of the following items how much it would help you in achieving better control of your diabetes (better glucose levels on blood tests):

Response options:

	Very helpful	Somewhat helpful	Not so helpful	Not helpful at all
To exercise (going to the gym, walking, etc.)	☐	☐	☐	☐
To have regular visits with a dietitian	☐	☐	☐	☐
Not to get upset (less stress)	☐	☐	☐	☐
To maintain normal body weight	☐	☐	☐	☐
To eat a diet appropriate for patients with diabetes	☐	☐	☐	☐
To take the pills or injections the physician has prescribed	☐	☐	☐	☐

Section 2: Dietary Patterns

Are you adherent to a specific dietary pattern?

☐ Vegetarian
☐ Vegan
☐ Keto
☐ Paleo
☐ Intermittent Fasting
☐ Calorie counting
☐ Point counting
☐ Other (please specify): ________________________

☐ No specific diet

Section 3: Sources of dietary education or guidance

Please name your sources of diabetes dietary education or guidance:

(Open-ended response - interviewer will document spontaneously mentioned sources)

Possible sources include:

☐ Family physician
☐ Diabetes specialist physician
☐ Nurse
☐ Dietitian
☐ Friend/neighbor/family member
☐ TV/radio/newspaper
☐ Social Media
☐ Lectures at the community center or at a clinic
☐ Newsletter by the health care organization
☐ Trainer or alternative therapist
☐ Other (please specify): ______________________
☐ I don't have any sources of dietary information

Section 4: Dietary consultation attendance and experience

Have you ever attended a dietary consultation? ▫ Yes ▫ No

If yes, please answer the following questions:

Patient-Reported Outcome Measures (PROMs)

Please answer Yes or No to the following questions about your experience with dietary consultations:

1. Did the dietitian speak your language?
   ☐ Yes ☐ No
2. Were the dietary recommendations adjusted to your culture and family context?
   ☐ Yes ☐ No
3. Were the recommended foods tasty?
   ☐ Yes ☐ No
4. Was the diet satiating (kept you full)?
   ☐ Yes ☐ No
5. Did the recommendations take into account your personal food preferences?
   ☐ Yes ☐ No
